# Role of RNA Domain Structure and Orientation in the Coxsackievirus B3 Virulence Phenotype

**DOI:** 10.1128/jvi.00448-23

**Published:** 2023-04-19

**Authors:** Lydia Phillips, William E. Tapprich

**Affiliations:** a Biology Department, University of Nebraska at Omaha, Omaha, Nebraska, USA; Instituto de Biotecnologia/UNAM

**Keywords:** 5′ untranslated region, coxsackievirus B3, enterovirus, internal ribosome entry site, RNA structure

## Abstract

Coxsackievirus B3 (CVB3) is an enterovirus that causes diseases such as pancreatitis and myocarditis in humans. Approximately 10% of the CVB3 RNA genome consists of a highly structured 5′ untranslated region (5′ UTR) that is organized into six domains and contains a type I internal ribosome entry site (IRES). These features are common to all enteroviruses. Each RNA domain plays a vital role in translation and replication during the viral multiplication cycle. We used SHAPE-MaP chemistry to generate secondary structures of the 5′ UTR from the avirulent strain CVB3/GA and the virulent strain CVB3/28. Our comparative models show how key nucleotide substitutions cause major restructuring of domains II and III of the 5′ UTR in CVB3/GA. Despite these structural shifts, the molecule maintains several well-characterized RNA elements, which allows persistence of the unique avirulent strain. The results shed light on the 5′ UTR regions serving as virulence determinants and those required for fundamental viral mechanisms. We used the SHAPE-MaP data to produce theoretical tertiary models using 3dRNA v2.0. These models suggest a compact conformation of the 5′ UTR from the virulent strain CVB3/28 that brings critical domains into close contact. In contrast, the model of the 5′ UTR from the avirulent strain CVB3/GA suggests a more extended conformation where the same critical domains are more separated. Our results suggest that the structure and orientation of RNA domains in the 5′ UTR are responsible for low-efficiency translation, low viral titers, and absence of virulence observed during infection by CVB3/GA.

**IMPORTANCE** Human enteroviruses, which include five different species and over 100 serotypes, are responsible for diseases ranging from mild respiratory infections to serious infections of pancreas, heart, and neural tissue. All enteroviral RNA genomes have a long and highly structured 5′ untranslated region (5′ UTR) containing an internal ribosome entry site (IRES). Major virulence determinants are located in the 5′ UTR. We present RNA structure models that directly compare the 5′ UTR derived from virulent and avirulent strains of the enterovirus coxsackievirus B3 (CVB3). The secondary-structure models show rearrangement of RNA domains known to be virulence determinants and conservation of structure in RNA elements known to be vital for translation and replication in the avirulent strain CVB3/GA. The tertiary-structure models reveal reorientation of RNA domains in CVB3/GA. Identifying the details of structure in these critical RNA domains will help direct antiviral approaches to this major human pathogen.

## INTRODUCTION

Coxsackievirus B3 (CVB3) is a positive-strand RNA virus in the genus *Enterovirus* of the family *Picornaviridae*. Infection by CVB3 causes pancreatitis, meningitis, and myocarditis in humans (1, 2). In fact, CVB3 is a leading infectious cause of myocarditis, which can lead to dilated cardiomyopathy (3). The CVB3 genome of approximately 7,400 nucleotides is organized into a coding region flanked by untranslated regions on both the 5′ and 3′ ends and has a 3′ poly(A) tail (4). The coding region is translated to yield a small peptide derived from an upstream open reading frame (ORF) (5) and a 2,185-amino-acid polyprotein that is processed by two viral proteases into 11 different viral proteins. A long and structurally complex 5′ untranslated region (5′ UTR) occupies the first 10% of the genome and contains RNA elements that form a type I internal ribosome entry site (IRES) responsible for recruiting canonical translation initiation factors, ribosomes, and noncanonical IRES transacting factors (ITAFs) (6, 7). These 5′ UTR RNA elements drive a cap-independent mechanism for translation initiation (8–10) and also recruit viral proteins and host proteins required for replication of the viral genome (11).

By orchestrating viral processes throughout the infection cycle, the 5′ UTR plays a pivotal role in determining multiplication efficiency and virulence. Unsurprisingly, multiple studies have shown that CVB3 virulence is modulated by RNA sequences and RNA elements in the 5′ UTR (12–16). Given the importance of the 5′ UTR in determining virulence, several approaches have been applied to produce and refine models of RNA secondary and tertiary structures in the region (9, 16–20). These models help to establish the structural basis for RNA function in the 5′ UTR. For understanding RNA contributions to virulence, CVB3 presents a distinct and unique advantage; two naturally occurring and closely related strains, one of which is highly virulent and one of which is avirulent. The virulent strain (CVB3/28) has been characterized as both pancreovirulent and cardiovirulent in mice (21). The avirulent strain (CVB3/GA) was isolated in 1956 from a clinical sample and has been extensively studied as a naturally occurring CVB3 strain that displays neither pancreovirulence nor cardiovirulence (22, 23). Sequence comparisons between CVB3/GA and a number of virulent clinical CVB3 isolates have documented over 60 nucleotide substitutions in the 5′ UTR (22). Direct comparison of secondary and tertiary structures in the 5′ UTRs derived from these strains presents an opportunity to understand the RNA elements that determine virulence in a clinically important enterovirus.

The primacy of structure-function relationships in many RNA species has driven the development of increasingly sophisticated tools for high-acuity structural analysis. Previous methods, such as base-specific chemical modification with dimethyl sulfate (DMS), 2-keto-3-ethoxybutyraldehyde (kethoxal), and 1-cyclohexyl-(2-morpholinoethyl) carbodiimide metho-*p*-toluene sulfonate (CMCT), were used to probe specific nucleotides in CVB3 (16, 20). These studies were useful in understanding nucleotide status as paired or unpaired and generated important secondary-structure models. However, more recent molecular probing methods, such as selective 2′-hydroxyl acylation by primer extension analysis (SHAPE) using *N*-methylisatoic anhydride (NMIA) and 1-methyl-7-nitroisatoic anhydride (1M7), allow for structural analysis at single nucleotide resolution with higher statistical accuracy using backbone-specific chemicals (24). The sensitivity of SHAPE experiments also provides valuable reactivity data which can be used for better detection of the less stable tertiary interactions (24–26). SHAPE analysis has already produced an updated secondary-structure model for the 5′ UTR of the virulent strain CVB3/28 (9). The most recently developed version of SHAPE analysis, which incorporates mutational analysis (SHAPE-MaP), has further improved sensitivity and accuracy (24, 27–29). Secondary structures derived from SHAPE-MaP analysis also provide optimization parameters for building three dimensional models of large RNAs using computational folding protocols such as 3dRNA v2.0 (30, 31).

In this study, we used SHAPE-MaP, TurboFold II, and 3dRNA v2.0 to generate secondary and tertiary models for 5′ UTR RNA from CVB3/28 and CVB3/GA. These models enable direct structural comparison of 5′ UTRs derived from virulent and avirulent viruses. At the secondary-structure level, the models show significant rearrangement in domain II, also called stem-loop II (SLII), a domain that is well known for involvement in the virulence phenotype in CVB3 and other enteroviruses (13, 15, 32, 33). Important differences in the secondary structure are also evident in domain IV. Tertiary-structure modeling reveals that these rearrangements have substantial impact on the orientation and positioning of many RNA domains in the molecule. Together, the results suggest that key sequence variations alter the positions of RNA structural domains that function in concert, thereby influencing efficiency of the 5′ UTR function and modulating virulence.

## RESULTS

### 5′ UTR sequence comparison.

**FIG 1:**
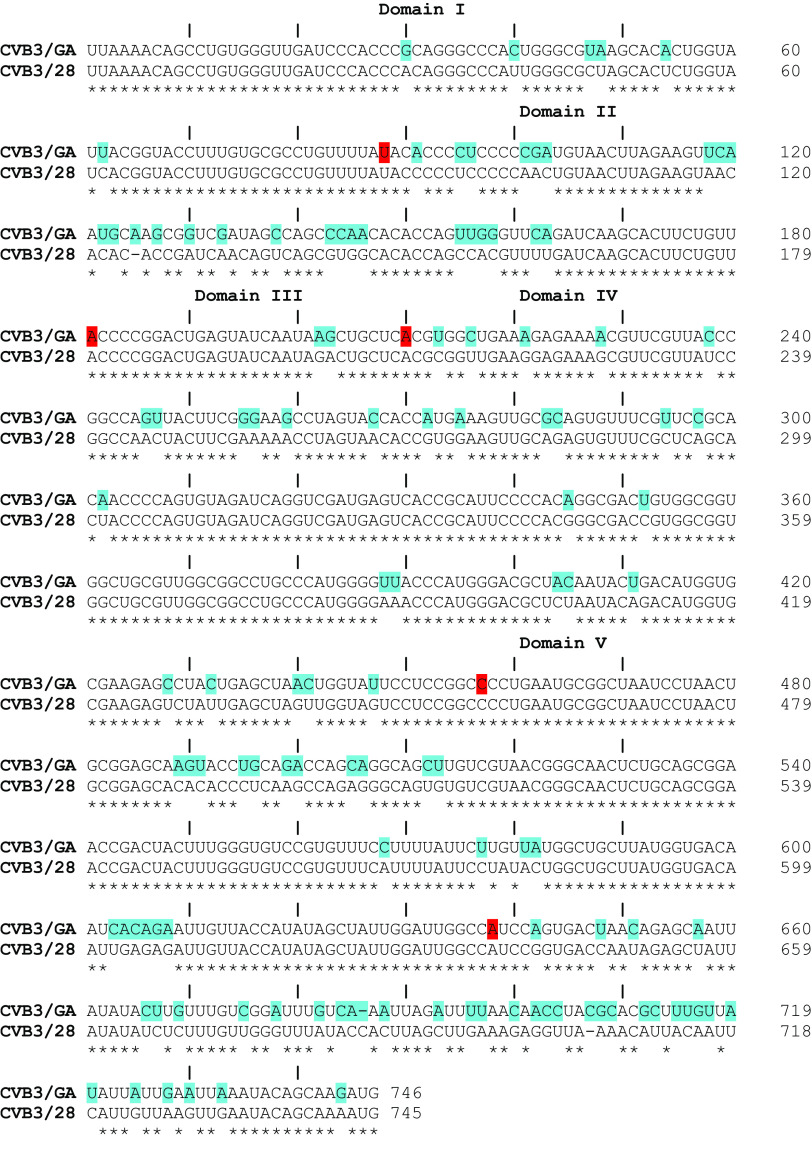
Sequence alignment of the 5′ UTR from CVB3/GA and CVB3/28. Blue highlighting shows the positions with nucleotide substitutions. Red highlighting shows the boundaries of each 5′ UTR domain in CVB3/28.

The 5′ UTR extends from the 5′ end of the genome to the start codon for polyprotein translation, position 743 in CVB3/28 and position 744 in CVB3/GA. A sequence alignment showing the positions of the 5′ UTR that differ between the two strains from the 5′ end to the polyprotein start codon is shown in Fig. 1. As shown in the figure, each of the RNA domains has nucleotide substitutions, with some domains being more highly conserved than others. From domains I to V, there are 85 positions that differ. Domains I and III have differences at 7% of the positions, domains IV and V have differences at 12% of the positions, and domain II has differences at 28% of the positions. In each domain, many of the nucleotide substitutions found in base-paired helical regions are swaps that create a GU base pair in one 5′ UTR where a GC or AU pair is present in the other strain. Similarly, there are several examples where nucleotide substitutions are compensatory and maintain base pairing.

### 5′ UTR secondary-structure models.

**FIG 2:**
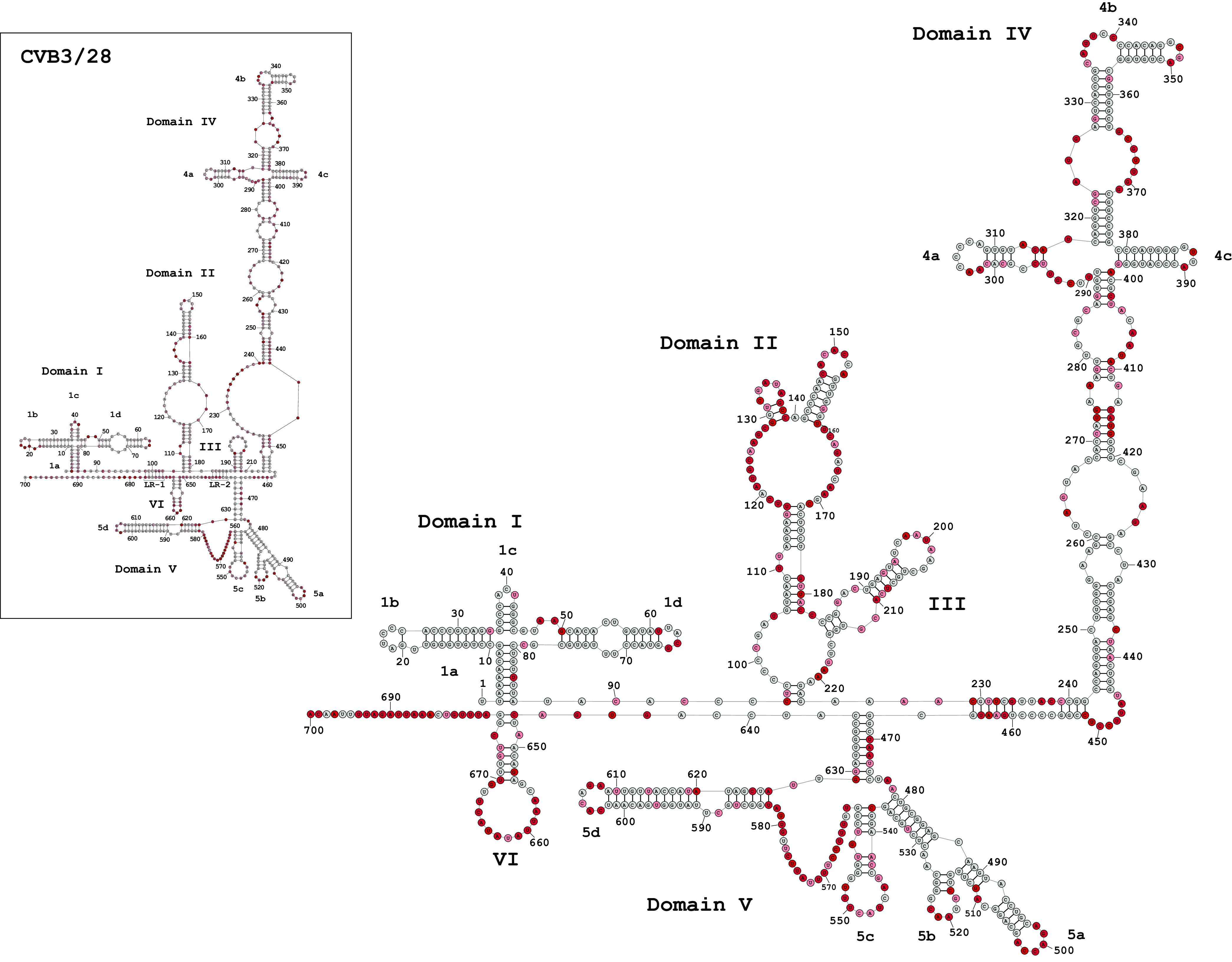
Secondary structures of CVB3 5′ UTR regions. 5′ UTR RNA from CVB3/28 and CVB3/GA was modified with 1M7 following the SHAPE-MaP protocol. ShapeMapper 2.0 and TurboFold were used to analyze and predict the secondary structures. The nucleotide shading indicates normalized reactivity values of <0.3 (gray), 0.3 to 0.7 (light red), and >0.7 (dark red).

We determined secondary structures for the 5′ UTR of the virulent CVB3/28 strain and the avirulent CVB3/GA strain using SHAPE-MaP and TurboFold II (Fig. 2). Three biological replicates of each strain were analyzed, and the results were 99% in agreement in base-paired positions. In previous work, we presented a secondary-structure model for CVB3/28 using analysis from SHAPE-CE and TurboFold II (9). The CVB3/28 model from our previous work was confirmed in all three replicates of our current SHAPE-MaP runs; therefore, we present the previously published structure of CVB3/28 in the inset of Fig. 2 for comparison to CVB3/GA.

The secondary-structure model derived from SHAPE-MaP analysis for CVB3/GA shows that the general domain organization is conserved compared to that of CVB3/28. However, nucleotide substitutions in each domain lead to secondary-structure changes, some of which are minor and some of which result in substantial reorganizations.

Domain I, commonly called the cloverleaf, has only six positions with nucleotide substitutions and shows a secondary structure in CVB3/GA that is nearly identical to that of CVB3/28. Two substitutions occur in base-paired positions that maintain the helix by swapping a GC pair with a GU pair. The remaining substitutions occur in single-stranded positions. The change of U to A at position 54 adds a Watson-Crick pair to stem-loop 1d and shortens a three-nucleotide symmetric internal loop to a two-nucleotide symmetric internal loop. Interestingly, the three-nucleotide symmetric internal loop of CVB3/28 has been shown by nuclear magnetic resonance (NMR) to form pyrimidine-pyrimidine noncanonical pairs (34) that adopt a helical structure.

**FIG 3:**
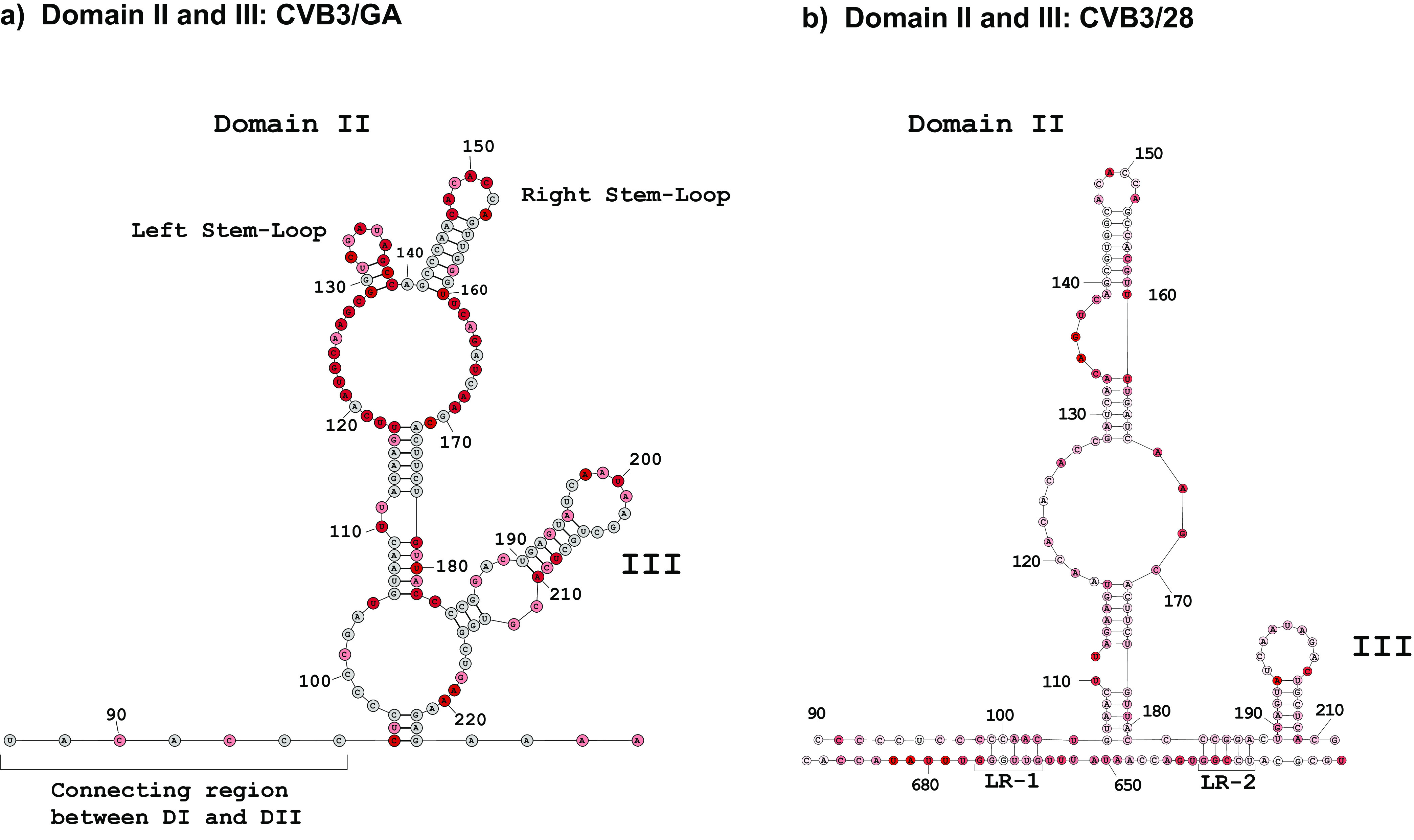
Comparison of secondary structures of domain II and III. 5′ UTR RNA from CVB3/28 and CVB3/GA was modified with 1M7 following the SHAPE-MaP protocol. ShapeMapper 2.0 and TurboFold were used to analyze and predict the secondary structures. (a) Secondary structure of superdomain II/III (95 to 224) of the CVB3/GA 5′ UTR. The single-stranded region leading from domain I (88 to 94) to the base of domain II is labeled as the connecting region between the two. The left stem-loop encompasses nucleotides 129 to 139. The right stem-loop encompasses nucleotides 141 to 160 and corresponds to the top stem-loop of domain II (139 to 160) in CVB3/28. Domain III includes positions 184 to 214. (b) Secondary structure of domains II (105 to 181) and III (189 to 210) of the CVB3/28 5′ UTR. LR-1 following the eight-nucleotide single-stranded connection region (90 to 97). LR-2 between domain II and domain III is also indicated. The nucleotide shading indicates normalized reactivity values of <0.3 (gray),0.3 to 0.7 (light red), and >0.7 (dark red).

Domains II and III show the greatest frequency of nucleotide substitutions and also display the greatest shift in secondary structure (Fig. 3). In CVB3/GA, rather than forming the single complex stem-loop closed by positions 105 and 181 as found in CVB3/28, domain II merges with domain III in CVB3/GA to form a “superdomain” closed by positions 95 and 224. Nucleotide substitutions at positions 95 (U to C) and 96 (C to U) on the 5′ side make the new closing helix possible in CVB3/GA. A three-nucleotide closing helix is admittedly short for a major RNA domain. Furthermore, positions 95 and 96 show moderate modification by 1M7 (0.47 and 0.32, respectively). Still, TurboFold II, which considers parameters of sequence comparison and energy minimization in addition to modification, includes this helix in each model generated from our nine independent SHAPE-MaP runs of CVB3/GA. A similar situation occurs in the domain II helix involving positions 105 to 109 and 178 to 182, where positions in the 3′ strand are moderately modified by 1M7. Again, all of our SHAPE-MaP runs for CVB3/GA resulted in models that had this helix. In this case, the CVB3/28 model has the same helix and the 3′ strand has positions that show 1M7 modification. Studies of 1M7 reactivity have demonstrated that several parameters of relative nucleotide positioning and nucleotide identity can increase or decrease expected reactivity (25, 35–37).

In the merged superdomain, the closing helix sits below relatively long single-stranded regions on both the 5′ and 3′ sides. The 5′ single-stranded region includes nucleotide substitutions at positions 101 to 103, which interrupt the pairs that form long-range interactions 1 (LR-1) in CVB3/28, also making the closing helix of the merged superdomain possible in CVB3/GA. The 3′ single-strand region emerges from the closing helix of the merged domain III. This region includes positions that are part of the domain IV closing helix in CVB3/28, so they have an influence on the secondary structure of both domains III and IV.

Interestingly, the five-base helix and six-base helix separated by a two-nucleotide bulge loop (positions 110 and 111) which closes domain II in CVB3/28 are also present above the merged domain III in CVB3/GA. In addition, the stem-loop that caps domain II in CVB3/28 is also present in CVB3/GA. The stem region of this apical stem-loop has 8 nucleotide differences between CVB3/28 and CVB3/GA, but all are compensatory. In the capping region of domain II in CVB3/GA, the apical stem-loop is joined by a second stem-loop closed by positions 129 and 139. This stem-loop in CVB3/GA, made possible by substitutions at positions 130 and 138, replaces a functionally important bulge loop from 134 to 138 in CVB3/28 that is highly conserved in enteroviruses (38).

Similarly, the stem-loop of domain III in CVB3/28 is also present as a capping helix of domain III in the CVB3/GA superdomain, but the long-range interactions (LR-1 and LR-2) of CVB3/28 are replaced by the domain III closing helix and domain II closing helix. Overall, domains II and III in CVB3/GA show substantial rearrangements in secondary structure compared to those domains CVB3/28, but several individual local secondary-structure elements remain unchanged.

**FIG 4:**
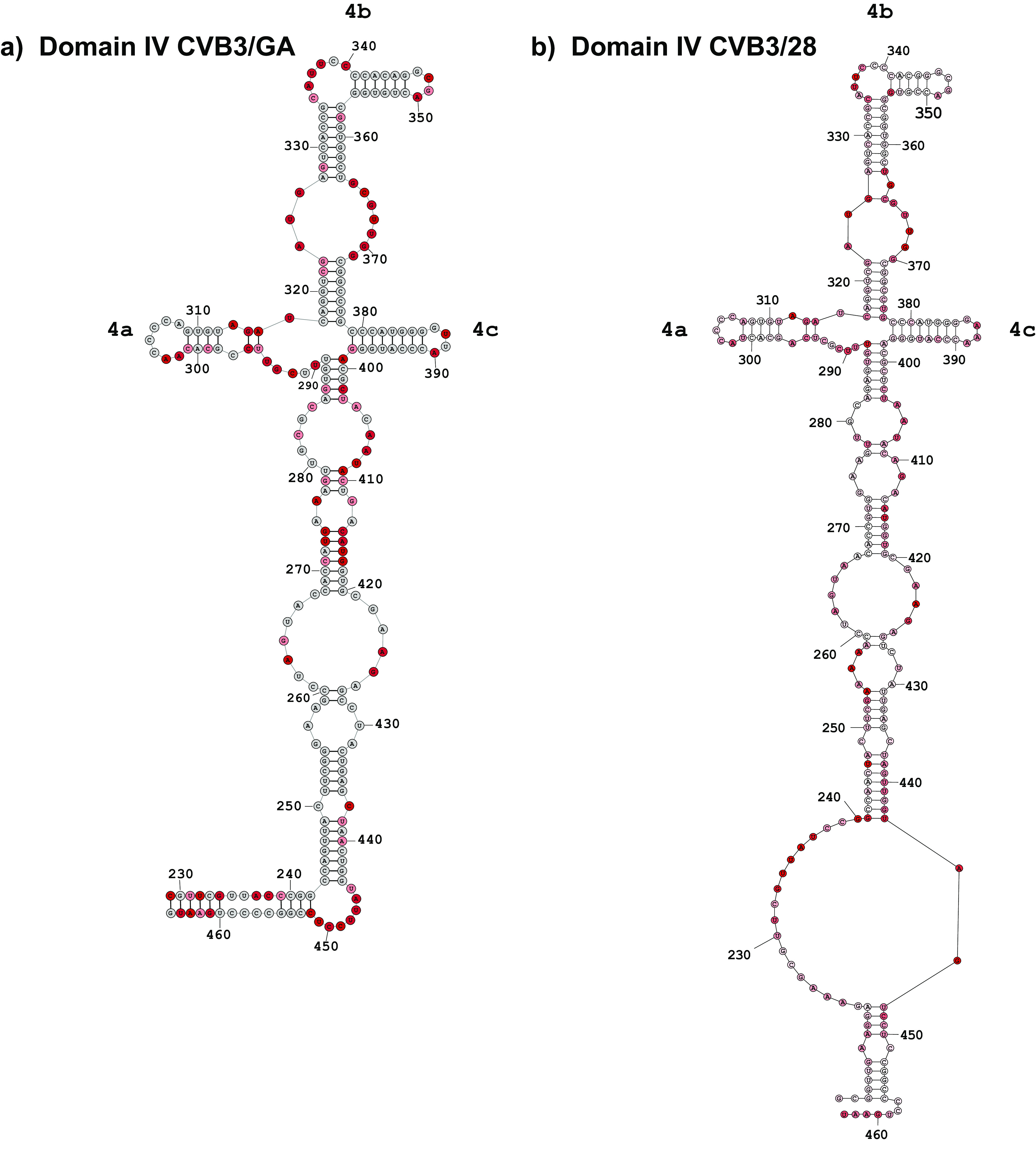
Comparison of domain IV secondary structure. 5′ UTR RNA from CVB3/28 and CVB3/GA was modified with 1M7 following the SHAPE-MaP protocol. ShapeMapper 2.0 and TurboFold were used to analyze and predict the secondary structures. (a) Secondary structure of domain IV of the CVB3/GA 5′ UTR (positions 229 to 465). (b) Secondary structure of domain IV of the CVB3/28 5′ UTR (213 to 456). Stem-loops 4a through 4c of the cruciform structure are labeled. The nucleotide shading indicates normalized reactivity values of <0.3 (gray), 0.3 to 0.7 (light red), and >0.7 (dark red).

When the secondary structures of domain IV in CVB3/28 and CVB3/GA are compared, the conserved structural elements far outnumber the differences (Fig. 4). As mentioned above, the position of the closing helix for domain IV begins at position 229 in CVB3/GA rather than position 213. This results in a long uninterrupted helical region at the base of the domain in CVB3/GA rather than a shorter helix that opens into a long asymmetric internal loop in CVB3/28. Beyond the closing helix, many of the differences in the long complex helix in the lower section of domain IV result in CVB3/GA forming more base-paired helices and fewer single-stranded regions than found in CVB3/28. There are several examples of compensatory substitutions that maintain helical regions in this lower section. Both CVB3/28 and CVB3/GA have a junction loop of three stem loops capping the domain. In both secondary structures, the C-rich single-stranded region around position 305 of stem-loop 4a and the C-rich single-stranded region around position 310 of stem-loop 4b as well as the tetraloops of stem-loops 4b and 4c are present. The sequence of the 4c tetraloop changes from GAAA in CVB3/28 to GUUA in CVB3/GA due to substitutions at positions 388 and 389.

**FIG 5:**
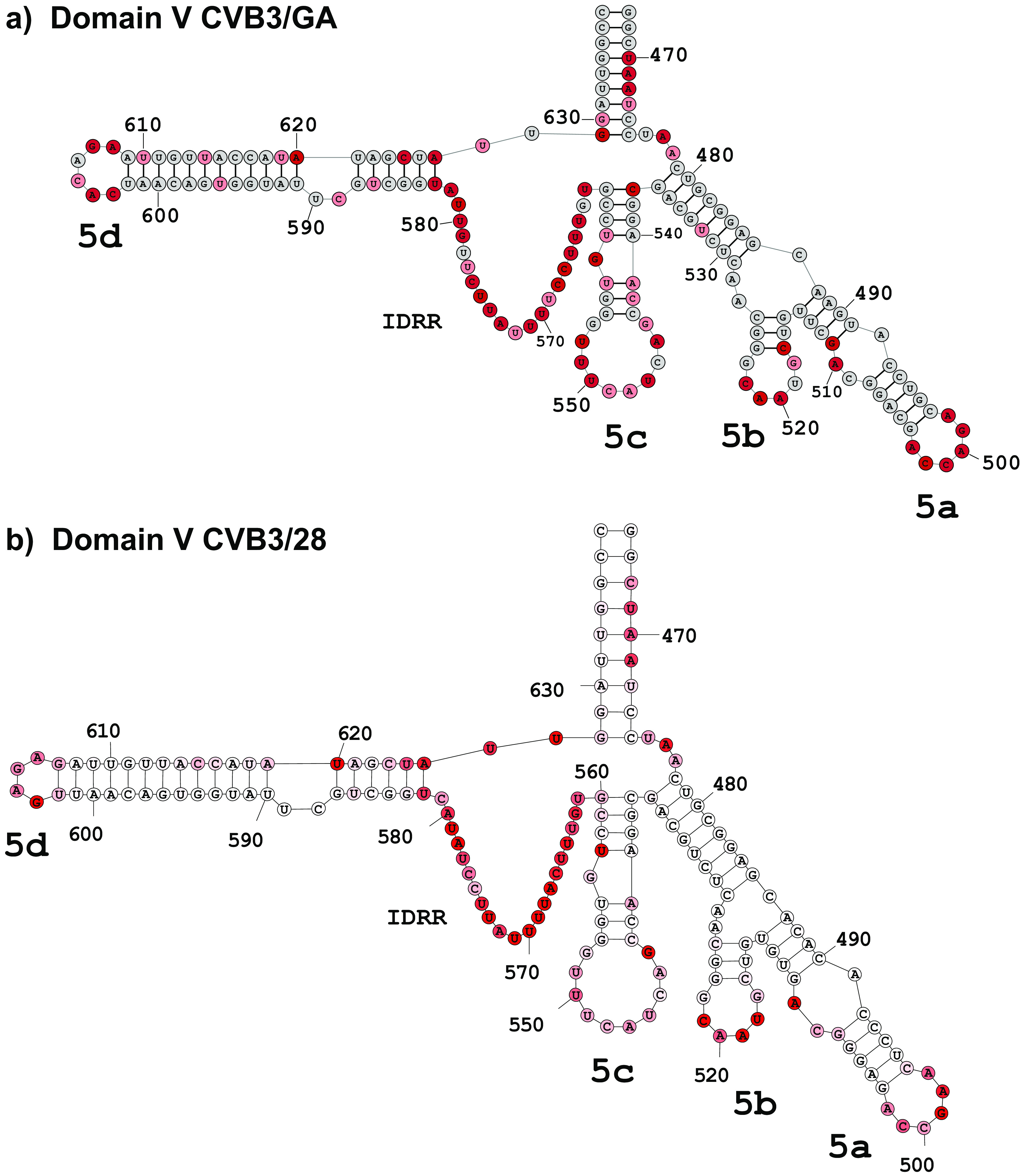
Comparison of domain V secondary structure. 5′ UTR RNA from CVB3/28 and CVB3/GA was modified with 1M7 following the SHAPE-MaP protocol. ShapeMapper 2.0 and TurboFold were used to analyze and predict the secondary structures. Stem-loop regions (5a to 5d) as well as the intrinsically disordered RNA region are labeled. The nucleotide shading indicates normalized reactivity values of <0.3 (gray), 0.3 to 0.7 (light red), and >0.7 (dark red). (a) Secondary structure of domain V in the CVB3/GA 5′ UTR. The IDRR of CVB3/GA includes nucleotides 562 to 582. (b) Secondary structure of domain V in the CVB3/28 5′ UTR. The IDRR of CVB3/28 includes nucleotides 561 to 581.

Our SHAPE-MaP results show that despite having 21 nucleotide substitutions, the very long and complex domain V is nearly identical in CVB3/28 and CVB3/GA (Fig. 5). The closing helix of 9 bp opens into a junction loop composed of three stem-loops (5ab, 5c, and 5d). The 20-nucleotide unstructured region between stem-loops 5d and 5c previously identified as an intrinsically disordered RNA region (IDRR) is present in both CVB3/28 and CVB3/GA. The only difference is the loss of a base pair in the stem below the hairpin loop of stem-loop 5d of CVB3/GA to make a 6-base hairpin rather than a tetraloop.

Secondary-structure interactions are necessarily defined by base-paired regions. The comparative models described above focus on changes that alter base pairing in each domain. However, single-stranded regions also adopt local orientations that depend upon base identity and each domain has sequence changes in positions that are single-stranded in both CVB3/28 and CVB3/GA. Most of the differences are single base changes, but a few are more extensive. Among the single base changes, a C-to-U alteration in stem-loop 1d of domain I converts a UACG tetraloop to a CACG tetraloop, and base changes at positions 568, 577, 581, and 582 are clustered in the IDRR of domain V. The longest string of base changes in a single-stranded region occurs from position 118 to position 127 in domain II, where CVB3/GA has UCAUGAG and CVB3/28 has AACCA-C. Again, domain II displays a significant alteration.

### 5′ UTR tertiary-structure models.

We used the 3dRNA v2.0 Web server and associated Web modules to analyze theoretical tertiary structures for the 5′ UTR of the virulent CVB3/28 strain and the avirulent CVB3/GA strain.

The 3dRNA v2.0 Web server includes a recommended step that uses direct coupling analysis data to generate energy probabilities of direct interactions. During the modeling process, which incorporates SHAPE-MAP data, five structures are generated and ranked by RNAScore. The RNAScore process is a novel scoring system that ranks the structures based on all nonhydrogen atoms, allowing for closer computational prediction of near native structures (39). A lower value from RNAScore represents a lower energy of folding, and higher probability. RNAScore values for the five lowest scoring models for CVB3/28 ranged from 26.11 to 26.21. RNAScore values for the five lowest scoring models for CVB3/GA ranged from 26.17 to 26.22. Overlaid tertiary structures for the five lowest scoring models of each strain showed very little deviation.

**FIG 6:**
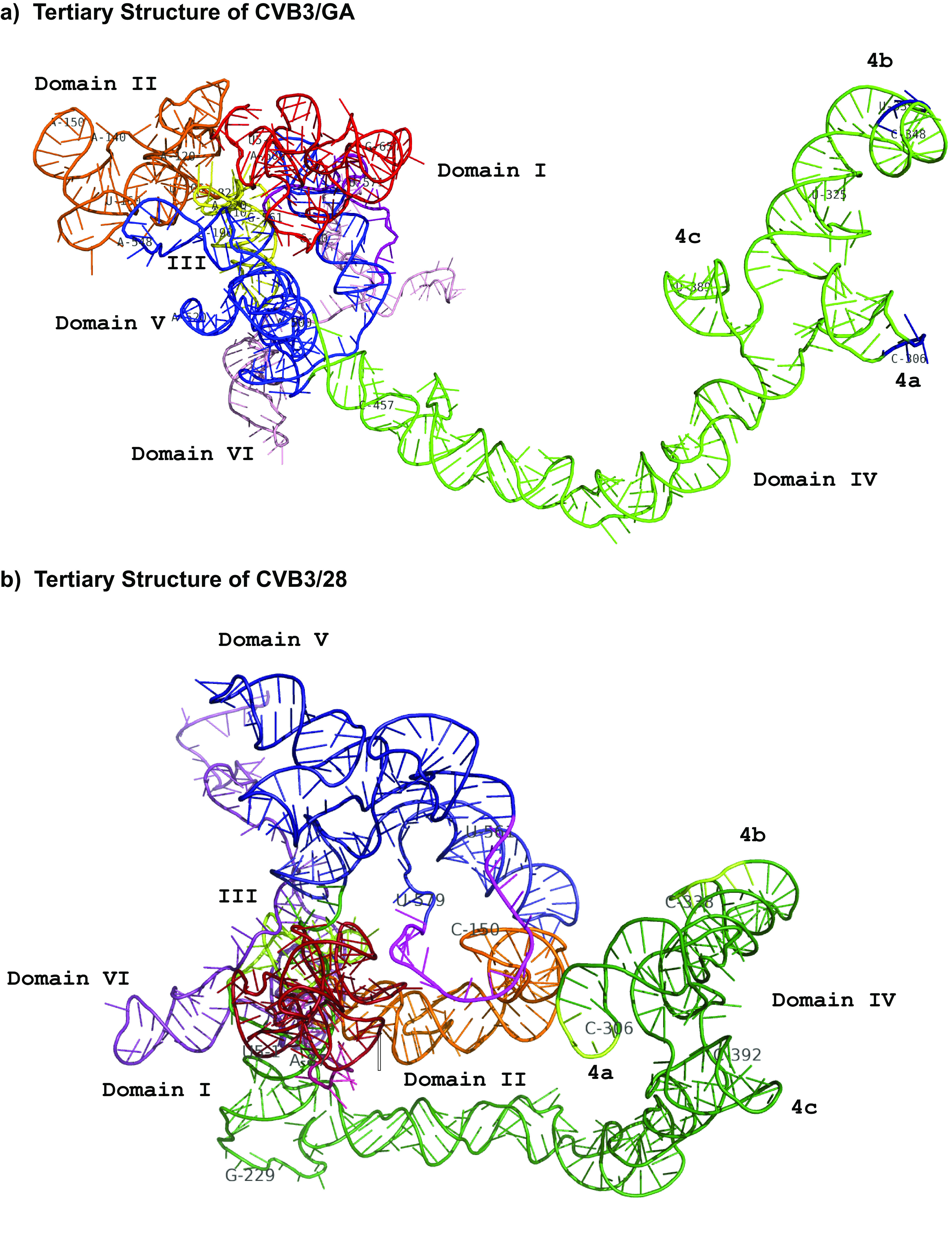
Tertiary structure models of CVB3 5′ UTRs generated by 3dRNA v2.0. Full-molecule renderings were generated using SHAPE-MaP secondary-structure data to inform 3dRNA v2.0 along with DCA. Tertiary molecules were visualized and colored by domain of interest in PyMOL: red, domain I; orange, domain II; yellow, domain III; green, domain IV; blue, domain V; purple, IDRR; pink, domain VI. Each molecule was oriented to place domain IV of each molecule in the same plane for easier comparison. (a) Tertiary structure of CVB3/GA. Poly(C) regions of domain IV (4a [304 to 307] and 4b [339 to 341]) are in blue. (b) Tertiary structure of CVB3/28. Poly(C) regions in domain IV (4a [304 to 308] and 4b [438 to 439]) are in light green.

The most obvious distinction between the tertiary models of the 5′ UTR in the two strains is the general compactness of CVB3/28 compared to the more dispersed conformation of CVB3/GA (Fig. 6). The models show that domain I in both CVB3/28 and CVB3/GA folds into a compact structure that is positioned in a central location of the overall molecule (Fig. 6). In CVB3/GA, domain II and domain IV emerge from opposite extremes of the extended conformation, whereas these two domains are adjacent in the more globular CVB3/28. Specifically, in CVB3/28, the hairpin loop around position 150 in domain II is very near to the cruciform capping domain IV as well as the IDRR (9) of domain V. In contrast, the hairpin loop around position 150 in CVB3/GA is along the edge of the folded molecule and distant from the cruciform of domain IV and the IDRR of domain V in CVB3/GA. In addition, the IDRR of domain V is rotated internally in CVB3/GA, and stem-loops 5ab, 5c, and 5d show closer association with the basal stem of domain IV, whereas the IDRR and stem-loops 5ab, 5c, and 5d of domain V are displayed on the periphery of the more globular 5′ UTR of CVB3/28, placing it in close proximity to the poly(C) regions of 4a and 4b in domain IV and the position 150 hairpin loop of domain II.

**FIG 7:**
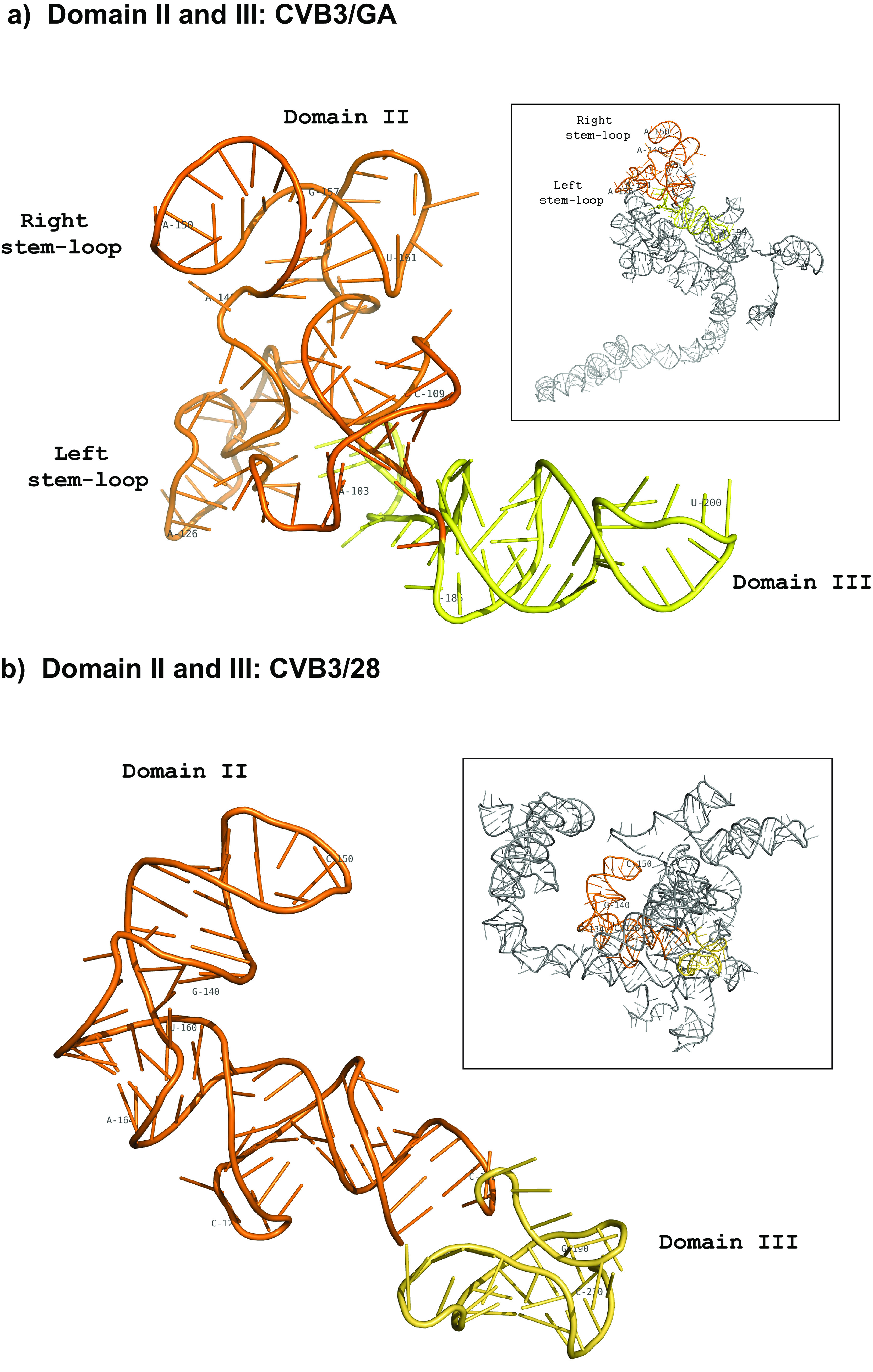
Tertiary structures of domain II/domain III. Full-molecule renderings were generated using SHAPE-MaP data secondary-structure data to inform 3dRNA 2.0 along with DCA. A single SHAPE-MaP structure was chosen based on the highest statistical quality checks from ShapeMapper 2.0. Individual domains were extracted from the full-molecule models using PyMOL and colored for clarity. (a) Tertiary structures of CVB3/GA superdomain II-III (95 to 224). (b) Tertiary structures of CVB3/28 domain II (105 to 181) and domain III (189 to 209). Domains were oriented to be within the same plane. Domain II is orange; domain III is yellow.

In CVB3/GA, domain II displays the two stem-loop pattern on the periphery of the molecule, but overall, superdomain II-III is more compact and globular than domain II of CVB3/28 (Fig. 7). As shown in the secondary-structure model, the well-conserved stem-loop surrounding position 150 is maintained in both CVB3/GA and CVB3/28. Both molecules also prominently display this conserved stem-loop.

**FIG 8:**
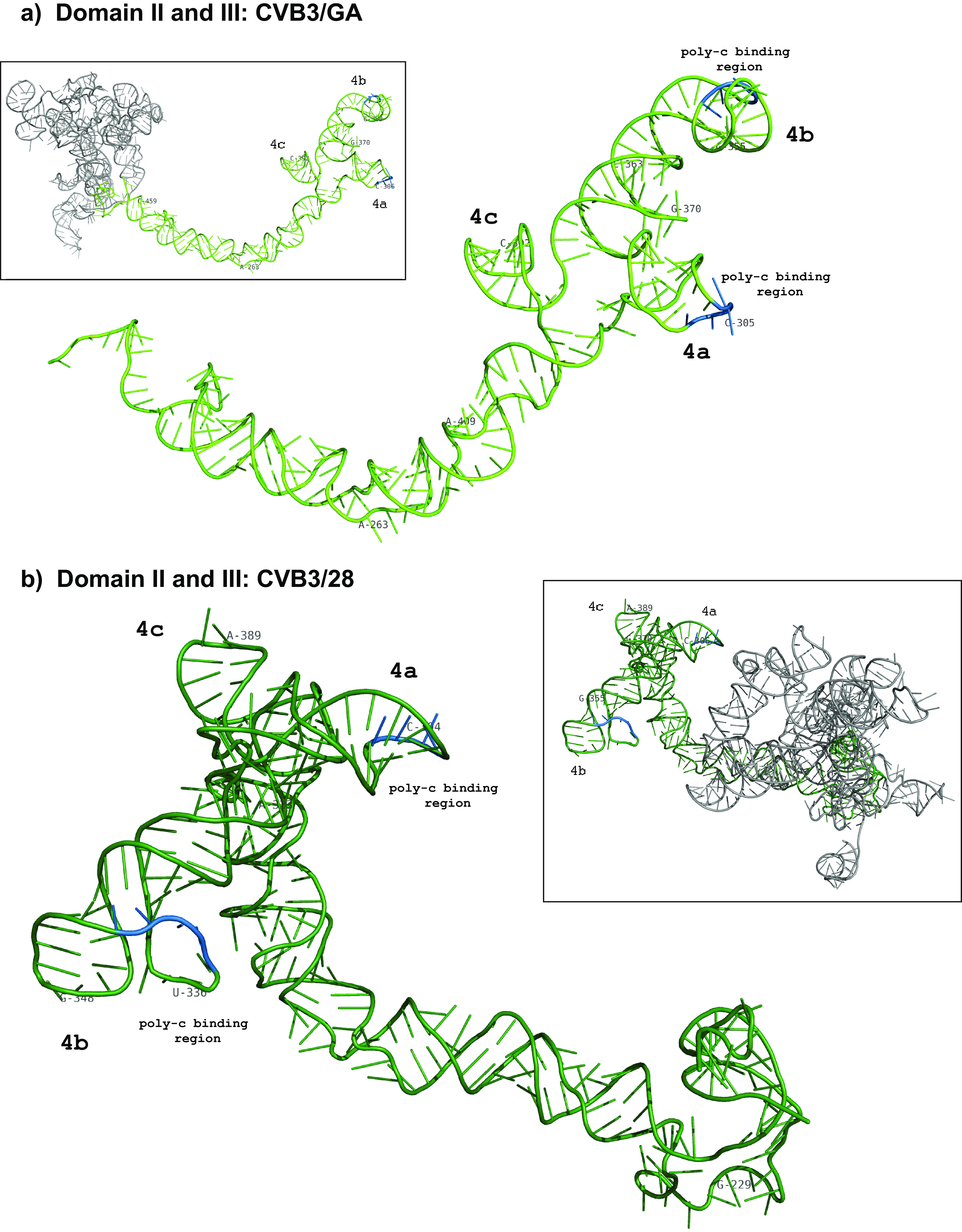
Tertiary structures of domain IV. Full-molecule renderings were generated using SHAPE-MaP data secondary-structure data to inform 3dRNA 2.0 along with DCA. A single SHAPE-MaP structure was chosen based on the highest statistical quality checks from ShapeMapper 2.0. Domain IV was again isolated from the full molecule in PyMOL. Each isolated domain IV was oriented to show as many of the vital regions as possible, such as the poly(C) binding regions. (a) CVB3/GA poly(C) regions in 4a (304 to 307) and 4b (339 to 341) are highlighted in blue. (b) CVB3/28 poly(C) regions 4a (303 to 306) and 4b (338 to 340) are also highlighted in blue.

The long basal stem of domain IV in both CVB3/28 and CVB3/GA extends outward from the more globular center and displays the cruciform junction loop on the end of the extension with similar overall orientation (Fig. 8). However, domain IV emanates from opposite poles of the globular center in the two molecules in a mirror image fashion. In CVB3/28, domain IV is rotated into close proximity to domain II. The C-rich loops of 4a (304 to 308) and 4b (438 to 439) are particularly close to the top stem region (146 to 150) of domain II. In CVB3/GA, the entirety of domain IV extends away from and on the side opposite to domain II.

**FIG 9:**
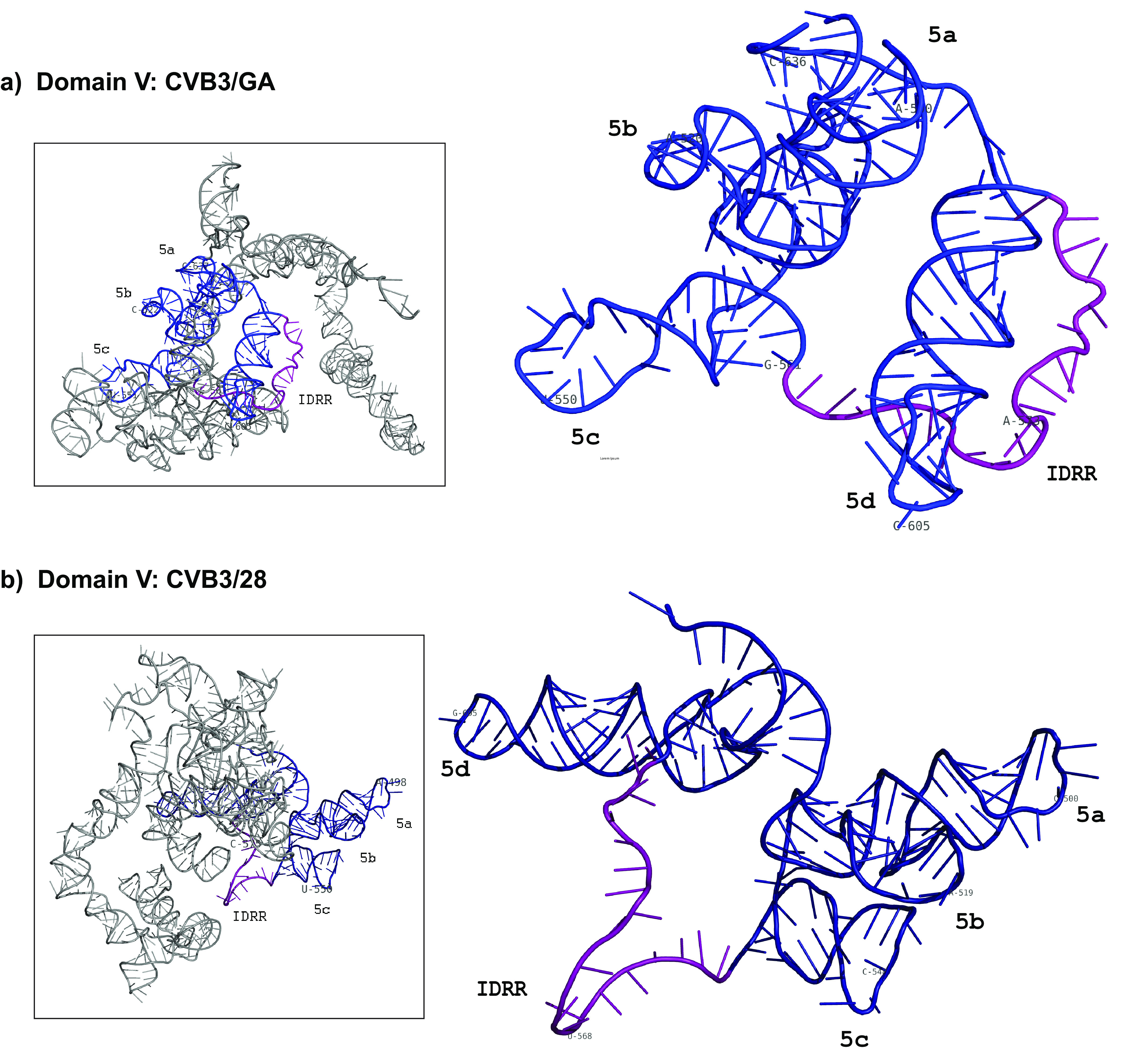
Tertiary structures of domain V. Full-molecule renderings were generated using SHAPE-MaP secondary-structure data to inform 3dRNA 2.0 along with DCA. A single SHAPE-MaP structure was chosen based on the highest statistical quality checks from ShapeMapper 2.0. Domain V was isolated from the full molecule in PyMOL. Stem-loops 5a to 5d are labeled. (a) CVB3/GA. the IDRR occupies 562U to 583A. (b) CVB3/28. The IDRR occupies 561U to 581C. Each domain V was oriented to focus on the IDRR (purple) conformation.

Domain V of CVB3/GA is in close contact with domain III and in the center of the most globular region of the molecule. The IDRR of the domain adopts a distorted helical conformation (Fig. 9). The beginning of the IDRR (positions 560 to 569) is nestled between stem-loop 5d and stem-loop 1b and internally rotated with the backbone facing out of the molecule. The central IDRR (570 to 578) has the backbone pointed away from the center and the bases directed inward. The end of the IDRR (579 to 582) shows the backbone rotated toward the center of the molecule and the bases oriented outward, causing the disordered region to appear as a distorted helix. In CVB3/28, domain V is displayed on one outer edge of the globular molecule. Stem-loops 5a to 5d and the IDRR radiate outward from a central point. In contrast to the zigzagged IDRR of CVB3/GA, the CVB3/28 IDRR exhibits a more extended pattern. The first few nucleotides of the IDRR (562 to 567) are oriented away from the central point of the domain. After a reverse turn in the backbone, the remaining nucleotides return to the central point. These nucleotides are located near the well-studied poly(C) region of stem-loop 4a and the position 150 stem-loop of domain II. The majority of the IDRR is in extended conformation and quite accessible.

## DISCUSSION

A long and highly structured 5′ UTR with an internal ribosome entry site (IRES) is a defining feature of the *Picornaviridae* genome (7). By circumventing the need for cap-dependent translation initiation, these simple viruses with relatively small genomes employ elements of RNA structure to overtake and dominate host cell gene expression. For picornaviruses with type I IRES elements, such as enteroviruses and rhinoviruses, the 5′ UTR consists of six structural RNA domains (9). Domain I is a cloverleaf structure of approximately 90 nucleotides that is primarily involved in genome replication (40) and has the viral protein Vpg covalently attached to the 5′ uracil. Domains II to VI have the RNA elements that together function as the IRES. The cloverleaf and the IRES are separated by a C-rich spacer of approximately 15 nucleotides (41).

Type I IRES elements require the assembly of canonical translation initiation factors (eIFs) and an array of host noncanonical proteins known as IRES transacting factors (ITAFs) to initiate translation (7, 42), the majority of which recognize and bind RNA elements in domains IV and V (43). The complex of eIFs and ITAFs interacting with the IRES enables the small ribosomal subunit to access the authentic start codons for the upstream coding region and the polyprotein coding region to accomplish cap-independent translation of viral proteins.

Critical RNA elements in the cloverleaf and IRES control efficient replication and translation, which in turn influence multiplication and virulence. For example, we showed previously that secondary-structure changes in the IRES accompany changes in CVB3 virulence (16). In the current study, we refined the secondary structure of the 5′ UTR from the avirulent CVB3/GA using SHAPE-MaP. While there are significant structural changes compared to the virulent strain CVB3/28, the similarity in local RNA structural elements between strains is striking. This similarity is particularly common in regions known to be directly involved in translation and replication functions, including regions where binding to host proteins is well understood, whether they be canonical initiation factors or noncanonical ITAFs.

Given the fundamental hierarchical role of RNA secondary structure in the folding pathway and final tertiary structure of the molecule (44, 45), there is good reason to make careful comparisons of secondary structure for homologous molecules that differ in function. This is particularly important for functional regions of viral genomes derived from strains that differ in virulence. CVB3 is ideal for this comparison because the virus presents the unique advantage of naturally occurring strains that differ greatly in virulence; an avirulent strain (CVB3/GA) and a virulent strain (CVB3/28) (22).

Several studies have examined sequence differences in the 5′ UTR between CVB3/GA and other pathogenic strains of CVB3 (16, 22). While sequence identities across the entire 5′ UTR are in the low 80th percentile in these comparisons, the highest frequency of sequence substitutions is clustered in the connecting region between domain I and domain II and in domain II (SLII). In the direct comparison between CVB3/28 and CVB3/GA, in the region encompassing domains II and III, 21% of the positions have sequence changes. In the remaining regions of the 5′ UTR, only 10% of the positions have sequence changes. Our SHAPE-MaP analysis shows that the large number of sequence changes in domains II and III result in a major reorganization of the secondary structure in CVB3/GA in this region. The reorganization includes merging domains II and III into a single superdomain and converting a conserved bulge loop-hairpin loop motif (38) into a two-hairpin-loop structure and also features a long string of consecutive nucleotide changes in a single-stranded region.

The major changes in secondary structure in domains II and III are joined by more limited changes that impact the basal helix of domain IV. By increasing the length of the domain IV closing helix and reducing the single-stranded regions, there may be reduced flexibility that leads to the extended orientation observed in the CVB3/GA tertiary model. Outside domains II, III, and IV, most secondary structures remain largely unchanged. Furthermore, many of the local RNA structural elements in domains II, III, and IV also remain unchanged. These results underscore the hierarchical folding of RNA elements within domains as well as domains themselves and also supports independent formation of RNA domains in the 5′ UTR (16).

Chimeric studies have mapped the cardiovirulence phenotype of CVB3 to the 5′ UTR (13) and specifically to SLII (12). Beyond the chimeric studies, little information is available about specific positions in domain II that may have particular influence on 5′ UTR structure and virulence. One limitation is the lack of naturally occurring avirulent CVB3 strains. Like CVB3/GA, the naturally occurring CVB3/CO strain is not cardiovirulent (23) and shows a disproportionate number of sequence substitutions in domain II compared to virulent strains. However, the positions that undergo substitution are not consistent between CVB3/CO and CVB3/GA.

Studies of the avirulent CVB3/GA show that the virus multiplies less efficiently and to lower titers in mouse hearts (23) and in murine fetal heart fibroblasts (22). Given the virulence phenotypes ascribed to domain II, we suggest that the reorganization of secondary structure in domains II and III in CVB3/GA largely underlies the changes in orientation of RNA domains that alter the efficiency of viral multiplication, thereby altering virulence. The persistence of secondary structure in surrounding domains and in local RNA elements of domains II and III highlights regions where fundamental RNA-RNA and RNA-protein interactions critical for translation and replication mechanisms are less tolerant to change.

We present theoretical tertiary-structure models of the 5′ UTR from CVB3/28 and CVB3/GA that were generated by the computational methods of 3dRNA v2.0 informed by our SHAPE-MaP results. Despite steady progress in both experimental methods and computational methods, determining tertiary structure in large RNA molecules remains a challenge (46), but efforts such as RNA-Puzzles (47) show that biologically relevant information is available in theoretical models. Still, interpretation of models for RNA molecules the size of the 5′ UTR must be approached with caution. Most 3D predictive software is incapable of accepting RNA longer than 500 nucleotides. 3dRNA v2.0 is the exception. The creators of 3dRNA v2.0 have estimated that smaller RNA species have root mean square deviation (RMSD) scores between 1.2 Å and 9.05 Å. Larger species have larger RMSD scores. Recent improvements to 3dRNA v2.0 were tested on riboswitches with known tertiary structure and lengths between 500 and 2,900 nucleotides (48). The 500-nucleotide riboswitch structure had an RMSD of 5 Å. Three of the four longer riboswitches (1,500 to 2,800 nucleotides) had RMSDs under 15 Å. The fourth longer riboswitch of 2,900 nucleotides had an RMSD of 98 Å. For our models of the 5′ UTR, the strictest parameters were chosen to eliminate as many false results as possible.

At the level of overall conformation, our tertiary models suggest that the CVB3/28 5′ UTR is much more compact than the CVB3/GA 5′ UTR. Even without dissecting the orientation of individual domains, a more compact organization is likely to bring functional elements of the 5′ UTR into closer proximity. Little direct experimental evidence is available to describe spatial relationships and orientation of 5′ UTR domains. Our theoretical tertiary-structure models serve as a starting point for exploring these three-dimensional relationships. By analyzing locations in the 5′ UTR known to bind ITAFs and canonical translation factors, there are strong arguments supporting orientations that bring some domains together.

Stem-loops b and d in domain I bind the ITAF poly(rC)-binding protein 2 (PCBP2) and viral protein 3CD, respectively (40, 49, 50). Stem-loop a and bulge loop b in domain IV bind PCBP2 (49), which also recruits the ITAF SRp20 (51). Domain V has interaction sites for eIF-4G, which also recruits eIF-4A (52) as well as the ITAF Unr (53–55) Importantly, the ITAF Unr binds with high affinity to both domain II and domain V in the context of the full-length 5′ UTR but binds with very low affinity to each individual domain (53), which suggests that these domains are positioned close together in 5′ UTRs that are most efficient. Based on the models we have generated, critical functional elements in the cloverleaf of domain I, the cruciform junction loop of domain IV, and the entirety of domain V are brought together in CVB3/28 with the critical apical hairpin loop of domain II nestled between. Thus, domain II in the CVB3/28 model is in a central position that could mediate efficient functional interactions among these domains and their binding partners. In contrast, domains I and II are on the periphery and on opposite sides of one end of the molecule in the model of CVB3/GA with domains IV and V oriented away from each other to form the other end of the molecule.

Biophysical methods such as NMR, small-angle X-ray scattering (SAXS), and cryo-electron microscopy (cryo-EM) have explored the tertiary structure of enteroviral 5′ UTR stem-loop D in domain I (34, 56–58) and the junction loop of domain IV (43). High-resolution models from these studies offer detailed information about stem-loop orientation and presentation of nucleotides with and without protein partners. Our tertiary-structure models for the 5′ UTR of CVB3/28 and CVB3/GA recapitulate the details of stem-loop orientation and nucleotide presentation in these regions. Stem-loop D in domain I and the junction loop of domain IV are known to interact functionally (49, 59). Again, these critical RNA elements are positioned very close to one another in the model of the CVB3/28 5′ UTR and are much more separated in the model of the CVB3/GA 5′ UTR.

The viral IRES presents an attractive therapeutic target (60). For enteroviruses, a morpholino oligomer targeting the IDRR in domain V reduced poliovirus titers in cell culture and protected mice from lethal doses of the virus (61) and an intercalating agent suppressed viral translation in cell culture (62). Detailed structural models of the 5′ UTR can provide the basis for directed strategies for development of therapeutic compounds. For example, a recent study shows that a small molecule targeting the bulge-loop of domain II causes changes in the structure of nucleotides in the loop and also reorients the tertiary structure of the domain by altering the position of helices above and below the loop. Binding of the small molecule decreases the efficiency of viral translation (63). The bulge-loop region of domain II is where the avirulent CVB3/GA 5′ UTR undergoes its most significant reorganization, transitioning from a bulge-loop to a stem-loop. As experimental and computational tools for determining RNA structure continue to advance, additional RNA elements that can serve as targets for antiviral development will be revealed.

## MATERIALS AND METHODS

### Viral plasmid construction.

Plasmids containing copies of full-length CVB3/GA and full-length CVB3/28 cDNA genomes were kindly provided by Nora Chapman and Steve Tracy of the Enterovirus Research Laboratory, University of Nebraska Medical Center. Plasmids were modified to include an upstream T7 promoter (TAATACGACTCACTATAGG) along with the ribozyme sequence (ATGAGGCCGAAAGGCCGAAAACCCGGTATCCCGGGTTC) inserted between the T7 promoter and the 5′ end of the 5′ UTR sequence. This construction enabled production of a 5′ UTR with the authentic 5′-end nucleotide.

### 5′ UTR plasmid DNA isolation and purification.

Plasmids were maintained in E. coli Sure cells. Plasmid DNA from both strains was isolated using a QIAprep Spin miniprep kit (Qiagen) according to the manufacturer’s protocol. CVB3/GA DNA was linearized with the restriction enzyme BssHII. Efficient transcription by T7 RNA polymerase requires a template with no overhang; therefore, DNA polymerase Klenow fragment (1 U) and deoxynucleoside triphosphates (dNTPs) (32 μM) were added to blunt the end of the linearized DNA caused by cleavage with BssHI. CVB3/28 plasmid DNA was linearized with the restriction enzyme Ecl136II, which generates a blunt end. Digested DNA was purified through phenol and phenol-chloroform extraction followed by ethanol precipitation.

### *In vitro* transcription.

The 5′ UTR RNA for CVB3/GA and CVB3/28 was produced with MEGAScript T7 transcription kit (Invitrogen), following the manufacturer’s instructions. Reactions were performed identically for both strains apart from the input of 10 μg of CVB3/GA template DNA and 4 μg of CVB3/28 template DNA due to low RNA production from CVB3/GA. The transcription reaction mixtures were incubated at 37°C for 4 h. Turbo DNase I (2 U) (Thermo Fisher) was added, and reaction mixtures were incubated at 37°C for an additional 30 min to remove the DNA template. Upon completion, RNA was extracted with equal volumes phenol-chloroform and chloroform, precipitated with isopropanol, and pelleted by centrifugation, and the pellet was washed with 70% ethanol. The final RNA product was purified using the MEGAclear transcription clean-up kit (Invitrogen), following the manufacturer’s protocol. RNA yield was quantified with a Qubit fluorometer (Invitrogen).

### SHAPE-MaP: selective 2′ hydroxyl acylation by primer extension and mutational profiling.

The SHAPE-MaP protocol of Smola et al. (64) was used for the acylation and reverse transcription steps. RNA folding for the modified and unmodified reactions was performed by diluting purified RNA to 10 pmol of RNA in 12 μL of RNase-free water. Folding experiments were done in duplicate. The diluted RNA was incubated at 95°C for 2 min before being transferred immediately to ice for a minimum of 2 min. While on ice, 6 μL of 3.3× folding buffer (333 mM HEPES, pH 8.0; 333 mM NaCl; 33 mM MgCl_2_) was added. RNA was folded during incubation at 37°C for 20 min. RNA modification was performed in a duplicate series of three reactions: a modification reaction, a no-reagent control (unmodified), and a denatured control. The modified and denatured control reaction mixtures were prepared with a final concentration of 1 mM 1M7 dissolved in DMSO, and the unmodified reaction mixture was prepared with neat DMSO. To prepare the modified and unmodified reaction mixtures, 9 μL of folded RNA was added to the respective tubes and incubated for 85 s at 37°C. The denatured control reaction mixture was prepared with 10 pmol of purified RNA in 3 μL of RNase-free H_2_O, 50% formamide, and 1× DC reaction buffer (500 mM HEPES, pH 8.0; 40 mM EDTA) before incubation at 95°C for 10 min. The denatured RNA was then added to denatured control reaction tubes containing a final concentration of 1 mM 1M7 in DMSO and incubated at 95°C for 1 min. The duplicate RNA samples from all three reactions were pooled and purified using RNeasy mini spin columns (Qiagen) according to the manufacturer’s protocol and quantified using the Qubit fluorometer (Invitrogen).

Reverse transcription was performed with 10 μL of modified, unmodified, and denatured control RNA with 10 ng of random nonamer primers (New England Biolabs) and incubated at 65°C for 5 min before being placed on ice. While on ice, 8 μL 1× MaP buffer (50 mM Tris, pH 8.0; 75 mM KCl; 10 mM dithiothreitol [DTT]; a 0.5 mM concentration of each dNTP; 15 mM MnCl_2_) was added to each tube and incubated at 25°C for 2 min. SuperScript II reverse transcriptase (1 U) was added and incubated at 25°C for 10 min and then at 42°C for 3 h. SuperScript II was inactivated by incubation at 70°C for 15 min before the samples were rapidly cooled on ice. Second-strand synthesis was performed with the NEBNext module following the manufacturer’s instructions. The subsequent double-stranded DNA (dsDNA) was purified from the synthesis reaction using PureLink PCR micro-spin columns (Invitrogen) according to the manufacturer’s instructions and eluted in 10 μL of RNase-free water. The dsDNA from each reaction was quantified using a Qubit fluorometer. The dsDNA samples were submitted to the Genomics Core Facility at the University of Nebraska Medical Center (UNMC) for sequencing using the randomer work-flow protocol from the developers of SHAPE-MaP (64). Library preparation and paired-end sequencing was performed using Illumina NexteraXT library preparation kits. Briefly, cDNA samples were fragmented and tagged in one step, and barcodes were added during PCR amplification. Library size distribution and molarity were evaluated with a bioanalyzer.

### ShapeMapper 2.

The sequencing data in FASTQ format was uploaded to the ShapeMapper 2 program according to the protocols designed by Siegfried et al. (29) to create the mutational profiles in the form of a .shape file. A configuration file was created for easy access to analysis parameters. The first of many “quality checking” steps performed automatic trimming by base call quality. The remaining reads with more than 25 nucleotides were then aligned with the Bowtie2 program. The Bowtie2 parameters were set to the highest sensitivity to detect single nucleotide mismatches and multiple deletions. The highest-quality base calls were used when read pairs disagreed. Insertions were not included in the alignments.

Due to the randomer primer usage, local realignment was also performed by Bowtie2 so that ambiguous alignments and deletions could be removed. Following the alignment, a reactivity profile was created based on mutation rate and individual nucleotide reactivities from the modified, untreated, and denatured samples. First the mutation rate of each nucleotide was recorded by calculating the mutation count of mismatches and unambiguously aligned deletions divided by the read count of that nucleotide location followed by the automatic calculation of individual nucleotide reactivity. The calculated reactivities were normalized to the denatured control samples. All quality control checks from ShapeMapper 2 (27) were met for all samples that were used for downstream structure prediction. A minimum of three biological replicates from SHAPE-MaP analysis were used to generate structural models.

### Structural prediction with TurboFold II.

Mutational profile data, in the form of the .shape file, were uploaded into TurboFold II software, embedded within the RNAstructure program. A three-sequence FASTA file of functionally homologous sequences was created for CVB3/GA, and a 12-sequence FASTA file was created for CVB3/28. The first sequence was the specific strain itself. Because CVB3/GA is unique, CVB3/0, a lab-created avirulent strain, and CVB3/28 were chosen as the sequences. CVB3/28 was chosen because of its close evolutionary relationship to CVB3/GA. For CVB3/28, the maximum number of functionally homologous sequences was chosen. Each of the 12 strains chosen exhibited cardiovirulence and pancreatic virulence like CVB3/28. The sequences were obtained from the NCBI nucleotide database (Table 1). A configuration file was created per the TurboFold II installation instructions from the RNAstructure page of the Mathews lab website (65). TurboFold II calculated all probable base pairs and maximized the sum of all probabilities to generate the structure. The three main parameters to control the suboptimal structures for TurboFold II are maximum percent energy difference, maximum number of structures, and window size. Default values for window size and maximum number of structures were used (66–68). For maximum percent energy difference, we used 10% to ensure that only the most probable structures were predicted. Rsample mode function, which considers the pseudo-free energy restraints by matching the structure prediction to the MaP data, was also engaged in TurboFold II to refine the sum of probability calculations. Three structures were generated representing each of the three passing SHAPE runs for both CVB3/28 and CVB3/GA and were in agreement for 99% of the base-paired positions.

**TABLE 1:** Functionally homologous sequences of CVB3 from NCBI

Strain	Sequence	NCBI accession no.
CVB3/GA	CVB3/GA	AY673831.1
	CVB3/0	AY752945.1
	CVB3/28	AY752944.2
CVB3/28	CVB3/28	AY752944.2
	CVB3/08TC170	KR362878.1
	CVB3/2679	KJ489414.1
	CVB3/Beijing0811	GQ141875.1
	CVB3/KM06	KJ020100.1
	CVB3/MCH	EU144042.1
	CVB3/Nancy	JN048468.1
	CVB3/NIV099741LV204	JX476168.2
	CVB3/Woodruff	U57056.1
	CVB3/CVB3SD2012CHN	JX976770.1
	CVB3/MKP	KJ025083.1
	CVB3/NIV0914321LV141P5	KR107055.1
	CVB3/NIV0923491LV157	JX476169.2

### Theoretical modeling tertiary interactions.

The TurboFold II CT file output containing the predicted nucleotide interactions was converted to dot-bracket notation (DBN file) with the Mathews lab Web server (https://rna.urmc.rochester.edu/RNAstructureWeb/Servers/ct2dot/ct2dot.html). A FASTA file of the reference sequence 5′ UTR of each strain along with secondary-structure data (from SHAPE-MaP), was subjected a direct coupling analysis (DCA) by the Xiao Lab DCA webserver (http://biophy.hust.edu.cn/DCA).

DCA uses multisequence alignment to predict tertiary interaction-based coevolutionary inferences, resulting in a direct interaction (DI) file. Next, the reference FASTA file, DBN file, and DI file were uploaded to the 3dRNA v2.0 Web server (http://biophy.hust.edu.cn/3dRNA). Distance geometry and energy minimization were selected. The resulting theoretical structures were evaluated with 3dRNAscore, which measures the energy distribution between nucleotides from the predicted structure. The lower the score, the higher the probability of accurate base pairing and tertiary interactions. The 3dRNA v2.0-generated .pdb file was loaded in PyMOL version 2.5 (Schrödinger, LLC [https://pymol.org/2/]) for visualization and false coloring (69).
